# In the Matter of a State Sanatorium for Indigent Consumptives

**Published:** 1901-01

**Authors:** 


### In the Matter of a State Sanatorium for Indigent Consumptives.

The following "brief" of the case mentioned was prepared at the suggestion of the Governor and sent to him Dec. 20th, ult.:

*Hon. Jos. D. Sayers, Governor, State of Texas.*

DEAR SIR: In compliance with your request we beg leave to submit the following:

1st. The climate of this State being beneficial for consumption, (and this being very generally known), attracts to the State numbers of indigent consumptives, physically unable to earn a living. There are certain localities in the State that are especially sought by them, and the burden of their care falls upon a few municipalities, and a few tax-payers are compelled to pay for a vast number who do not belong to them by right of citizenship. This, in our opinion, is unjust, and we see no reason why there should not be an equal distribution of taxation for this purpose over the entire State.

2nd. The contagiousness of tuberculosis is now recognized. The germ of the disease, tubercle bacillus, is found chiefly in the sputum of consumptives, but also in cattle furnishing food and milk. The disease is communicable from one human being to another, from human to animal, and *vice versa.* It is one of the most fatal of the contagious diseases, and causes one death in every six deaths from all causes.

The only measure from which we can expect any relief is PREVENTION. As matters now stand, this is impossible. A general quarantine is out of the question; they cannot be prevented from entering the State, nor sent back when discovered. Hence, the only thing to do is to care for in the most humane way, those so unfortunate as to be dependant upon charity.

Every hospital, poorhouse and asylum in this State has tuberculous patients, and the death records no doubt show that consumption is the cause of the greatest number of deaths in them. Many of the consumptives in the hospitals and poorhouses are wanderers who have started on a pilgrimage in search of health, and are helped from town to town until they can no longer travel, when they find refuge in hospitals or poorhouses and remain there until death claims them, or until helped to some other town in the State. It is indeed pitiful to see these poor people seeking health, and yet doomed to confinement in a hospital ward or illy arranged poorhouse, *mingling with and endangering the lives of other inmates* of these institutions.

3rd. Aside from the question of humanity, it is a positive source of danger to the public health to have consumptives going over the State expectorating tons of tuberculous sputum, thus propagating the deeds of this disease in human and cattle.

4th. The question naturally suggests itself, that if an institution for the care of indigent consumptives were established, would it not bring a great many to the State who would not otherwise come?

We believe not, because they would be prevented from roaming from place to place, and it would soon be learned that when they became wards of charity, they would be sent to the institutions for indigent consumptives, practically quarantining them, and only those with means for their care, and not paupers, would come to Texas.

As a basis upon which to estimate the cost for the care of consumptive patients, the following data is submitted:

From June 1, 1898, to January, 1900, there were admitted into the city hospital of San Antonio, 191 consumptives, of which number eighty-five died. The number of days of treatment was 5296, at a cost of sixty cents a day, or $3177.60; burial expenses, $360. It may be safely estimated that it cost the county of Bexar in care, charity contributions, railroad fares, etc., another $3177.60, making a total of $7085.20, or an average of $353.76 per month.

Public health, if not philanthropy, should prompt us to use every means in our power to prevent the spread of this disease. Data obtainable where death records are kept show that the disease is on the increase in this State amongst those who have lived here three years and over. It is due public health that our people be protected, and instead of municipalities being taxed, these cases should become the wards of the State and isolated in salubrious parts of the country, and in an institution built and equipped with the object of restoring them to health, or making them comfortable during life.

If established, the institution after a short while could be made to an extent self-supporting, as many of the invalids would be able to work and be benefitted thereby.

Following is a summary of our reasons for recommending the establishment and maintenance of a home for indigent consumptives:

1st. That it would control to a great extent the spread of the disease.

2nd. That it would afford a comfortable home, and instead of having consumptives scattered all over the State, they would be isolated.

3rd. That it would cost the tax-payers less to support the institution, and the money so expended afford the invalids more comfort than they could get in a hospital or poorhouse.

4th. It would afford many a chance of regaining health which hospitals and poorhouses cannot do under existing circumstances.

5th. That it is a reflection upon civilization and a reckless disregard of public health to allow indigent consumptives to go from place to place in search of health.

6th. That these institutions once established and operated, would soon collect these circulating carriers of contagion.

We wish it distinctly understood that this is not a crusade against those who are unfortunately suffering from tuberculosis. It is far from it. But it is urged as a rational sanitary measure in the interest of the public health—a protection to every community and every family and every individual against a most insidious and deadly contagion to which all are exposed; and incidentally, as a humane measure.

Respectfully submitted,
F. Paschal, M. D.,
M. M. Smith, M. D.,
D. R. Fly, M. D.,
F. E. Daniel, M. D.,
of the Committee.

There are other matters which would have been mentioned in the above, except for the necessity of making it brief; for instance, data of State hospitals in other States.

